# Accelerated hermaphrodite maturation on male pheromones suggests a general principle of coordination between larval behavior and development

**DOI:** 10.1242/dev.202961

**Published:** 2024-07-08

**Authors:** Denis F. Faerberg, Erin Z. Aprison, Ilya Ruvinsky

**Affiliations:** Department of Molecular Biosciences, Northwestern University, Evanston, IL 60208, USA

**Keywords:** Larval development, Behavior, Pheromones, Social signals, Coordination, *C. elegans*

## Abstract

Environment in general and social signals in particular could alter development. In *Caenorhabditis elegans*, male pheromones hasten development of hermaphrodite larvae. We show that this involves acceleration of growth and both somatic and germline development during the last larval stage (L4). Larvae exposed to male pheromones spend more time in L3 and less in the quiescent period between L3 and L4. This behavioral alteration improves provision in early L4, likely allowing for faster development. Larvae must be exposed to male pheromones in late L3 for behavioral and developmental effects to occur. Latter portions of other larval stages also contain periods of heightened sensitivity to environmental signals. Behavior during the early part of the larval stages is biased toward exploration, whereas later the emphasis shifts to food consumption. We argue that this organization allows assessment of the environment to identify the most suitable patch of resources, followed by acquisition of sufficient nutrition and salient information for the developmental events in the next larval stage. Evidence from other species indicates that such coordination of behavior and development may be a general feature of larval development.

## Teaser

**Highlighted Article:** In larvae, foraging behavior and development are coordinated; male pheromones can modulate both, accelerating the onset of reproductive maturity in the opposite sex.

‘Life is what happens to us while we are making other plans.’

Allen Saunders (popularized by John Lennon)

## INTRODUCTION

In most animal phyla, larvae bridge embryogenesis and adulthood (Formery and Lowe, 2023). Larvae grow, and acquire adult morphology and function while navigating their habitats and engaging in complex behaviors. Life in variable environments demands that larval development be plastic (Moczek et al., 2011; Ragsdale et al., 2013; Simpson et al., 2011) and coordinated with behavior. Understanding how this is achieved is required for a comprehensive description of development in natural habitats.

**Fig. 1.**
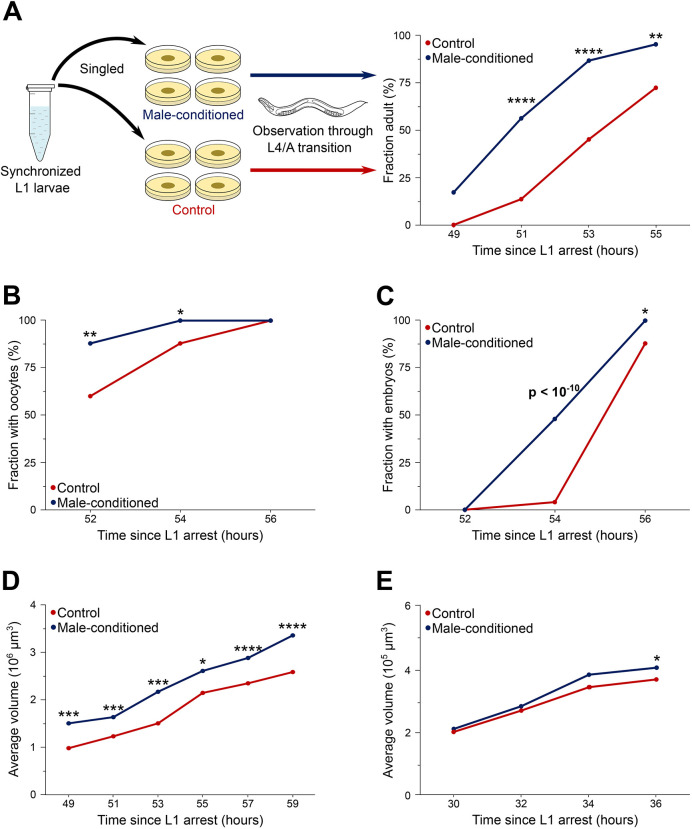
**Male pheromones accelerate growth and development of *C. elegans* hermaphrodite larvae.** (A) Larvae on male-conditioned plates (blue line) achieved adult vulva morphology earlier than controls (red line). (B,C) Frequency of individuals with at least one oocyte (B) and embryo (C). (D,E) Average volume of hermaphrodites in early adulthood (D) and mid-late L3 (E). *P<0.05, **P<0.01, ***P<0.001, ****P<0.0001. Additional data in Fig. S1. See Table S1 for sample sizes and statistical analyses.

Social environment is one type of external influence that can alter development (Baud et al., 2017). For example, *Caenorhabditis elegans* hermaphrodite larvae reach adulthood faster in the presence of male pheromones than on control or hermaphrodite-conditioned plates (Fig. 1A). This was shown in several previous studies in which we used time to adult vulva morphology (Figs S1A, S2A) as a measure of combined duration of larval development (larval stages L1-L4); we also showed that acceleration primarily affected the last larval stage (L4) (Aprison and Ruvinsky, 2016; Burkhardt et al., 2023; Ludewig et al., 2019). The ∼2 h (∼20%) average shortening of L4 on male pheromones could be detected even though there is considerable (>6 h) difference between the slowest- and the fastest-developing worms even under control conditions (Faerberg et al., 2021; Filina et al., 2022; Mata-Cabana et al., 2022; Stojanovski et al., 2022).

We initiated this study to address three questions raised by our previous work: are multiple aspects of late larval development coordinately accelerated? When does the acceleration start? Are any traits other than larval development altered by the male pheromones?

## RESULTS AND DISCUSSION

### Male pheromones cause coordinated acceleration of development around the L3-to-L4 transition in *C. elegans* hermaphrodite larvae

Production of oocytes (Fig. 1B; Fig. S1B) and embryos (Fig. 1C; Fig. S1C) started ∼2 h earlier on male-conditioned plates (MCP), indicating that multiple aspects of reproductive development were accelerated about as much as the vulva. During larval development, *C. elegans* hermaphrodites increase in volume ∼100-fold (Nyaanga et al., 2022; Stojanovski et al., 2022; Uppaluri and Brangwynne, 2015). We found that by the larva-to-adult transition, worms on MCP were larger than the controls (Fig. 1D; Fig. S1D). The accelerated growth was isomorphic (Fig. S1E) and advanced by the same ∼2 h as the development of the reproductive system. Divergence in size between control and MCP worms became evident at ∼34 h (Fig. 1E; Fig. S1F).

**Fig. 2.**
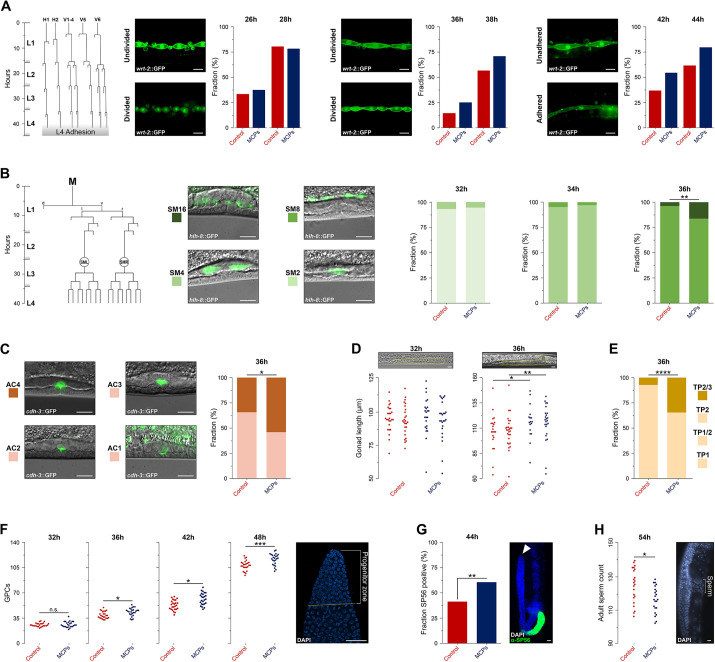
**Male pheromones coordinately accelerate late larval development in hermaphrodites.** (A) Fractions of larvae that initiated L3 seam cell division (26-28 h), L4 division (36-38 h) and L4 adhesion (42-44 h). (B) Fractions of larvae with indicated numbers of sex myoblast cells (M.vlpaa and M.vrpaa). Lineage diagrams and molting times (grey boxes) in A and B are from Sulston and Horvitz (1977). (C) Fractions of anchor cell ‘stages’ as indicated in images on the left. (D) Gonad (outlined with yellow dotted line) lengths measured at midline. Anterior arms on the left, posterior on the right. (E) Frequencies of phases of gonad turning. See Fig. S2E for staging. (F) Numbers of germline precursor cells. Dashed yellow line indicates the boundary of the progenitor zone. (G) Fraction of gonad arms expressing (at 44 h) SP56, an early marker of sperm differentiation. White arrowhead marks the distal end of the gonad. (H) Number of self-sperm in posterior gonads of adult hermaphrodites. In panels D, F, and H, each dot represents measurements/counts from one gonad arm. *P<0.05, **P<0.01, ***P<0.001, ****P<0.0001. Scale bars: 10 μm. Additional data in Fig. S2. See Table S1 for sample sizes and statistical analyses.

We next tested whether other developmental processes in mid-to-late hermaphrodite larvae accelerated on MCP: (1) divisions and adhesion of hypodermal seam cells (Fig. 2A; Fig. S2B), (2) sex myoblast divisions (Fig. 2B; Fig. S2C), (3) morphological transformations of the vulva anchor cell (Fig. 2C; Fig. S2D), (4) gonad growth and turning (Fig. 2D,E; Fig. S2E), (5) expansion of the population of germline progenitor cells (Fig. 2F), (6) sperm differentiation (Fig. 2G), and (7) sperm-to-oocyte switch (Fig. 2H). All of these processes were accelerated on MCP.

The accelerated expansion of the larval germline (Fig. 2F) is qualitatively different from the increased germline proliferation on the male pheromone ascr#10, because the latter only affects egg-laying adults, not larvae (Aprison et al., 2022; Aprison and Ruvinsky, 2019). Also, earlier termination of spermatogenesis (Fig. 2H) reveals environmental plasticity of the sperm-to-oocyte switch and argues that the hermaphrodite reproductive system responds to the signals that indicate the presence of males by redirecting resources into oogenesis (Angeles-Albores et al., 2023; Aprison et al., 2022).

The data in Figs 1 and 2 answer the first two of our three questions. First, male pheromones coordinately speed up somatic and germline development, resulting in earlier maturation of hermaphrodites. Growth is similarly hastened consistent with the previously reported coupling of the growth rate and developmental tempo (Gritti et al., 2016; Stojanovski et al., 2022).

Second, evidence from multiple tissue-specific markers and growth are highly concordant – acceleration starts shortly before 36 h. By this time, all processes we examined were more advanced on MCP, whereas only growth appeared to be advanced at an earlier time point (Fig. 1E). Cell divisions in several lineages occur at the onset of a larval stage (Sulston and Horvitz, 1977). At 36 h, ∼10% of control larvae had an L4-characteristic number of seam cells (Fig. 2A) and <10% had an L4-characteristic number of sex myoblast cells (Fig. 2B), indicating that under our experimental conditions the fastest developing larvae probably entered L4 at around 35 h. To summarize, male pheromones coordinately speed up multiple aspects of larval development to yield functional, mature hermaphrodites ∼2 h earlier. The acceleration starts around the L3-to-L4 transition.

### Male pheromones influence commitment to the lethargus between L3 and L4 stages

**Fig. 3.**
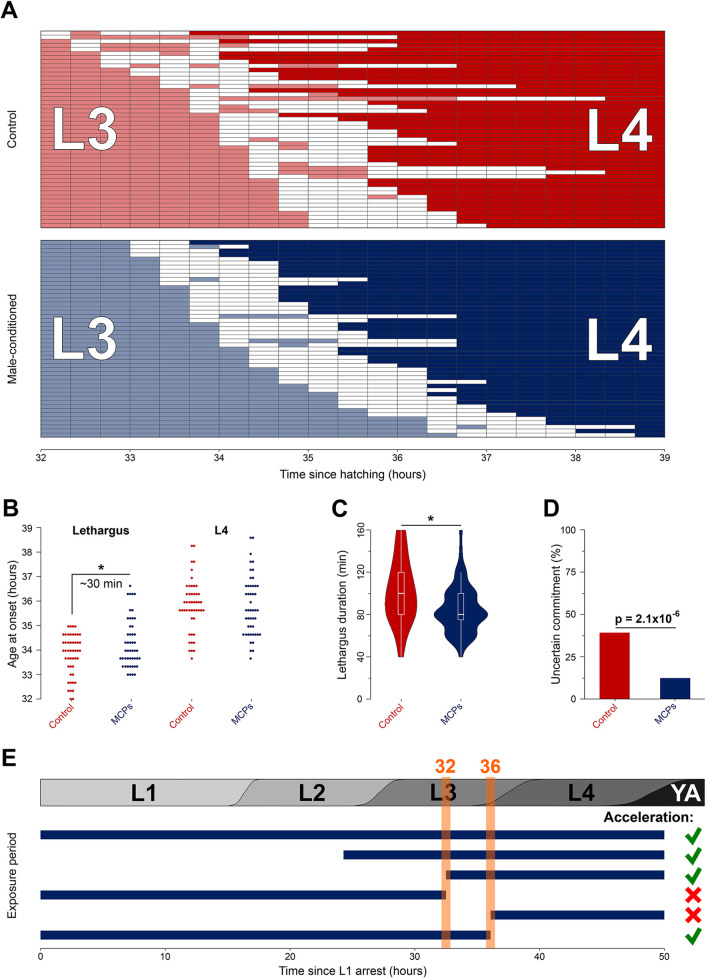
**Male pheromones and developmental time.** (A) Pharyngeal pumping activity, assayed at 20 min intervals, of control (red) and MCP (blue) hermaphrodite larvae (one larva=one row) around the L3-to-L4 transition. Color indicates pumping; white intervals indicate non-pumping, presumed lethargus. Sorted by the first observation period when pumping was not detected. This experiment was started with a timed egg lay. (B) Age of worms at first entry into presumed L3/L4 lethargus (left) and at the onset of L4 (right). Each dot represents one individual. (C) MCP larvae spent less time in the L3/L4 lethargus. Plot shows median values (middle bars) and first to third interquartile ranges (boxes); whiskers indicate approximately 1.5× the interquartile ranges. (D) Fractions of individuals that resumed pumping before re-entering lethargus. (E) Sensitivity window to male pheromones. The grey bar above shows approximate durations of larval stages. Sigmoidal transition boundaries between larval stages represent variability in developmental rates. Horizontal blue bars represent exposure to male pheromones. *P<0.05. Additional data in Fig. S3. See Table S1 for sample sizes and statistical analyses.

To better understand the onset of developmental acceleration, we next focused on the events in late L3/early L4 stages. In *C. elegans*, larval stages are separated by periods of behavioral quiescence, called lethargus, that typically last 1-2 h (Singh and Sulston, 1978). We considered three explanations for acceleration: (1) earlier termination of L3, (2) shorter lethargus or (3) shorter L4. To distinguish between these scenarios, we monitored pharyngeal pumping during the L3-to-L4 transition (Fig. 3A), because pumping ceases during lethargus (Raizen et al., 2008; Van Buskirk and Sternberg, 2007). We report five findings. First, hermaphrodite larvae on MCP on average exited L3 later than controls (Fig. 3B; Fig. S3A). Second, duration of quiescence preceding L4 was shorter on MCP largely because there were fewer long (>100 min) lethargus episodes (Fig. 3C). Third, the onset of L4 was indistinguishable between MCP and control plates (Fig. S3A,B). Fourth, ∼50% of larvae exited L3 at 34 h and entered L4 at 36 h (Fig. S3A), consistent with the estimates based on developmental markers (section above). Finally, fewer MCP larvae had an episode of inactivity followed by the resumption of pumping and then another episode of quiescence before emerging as an L4 (Fig. 3D).

These results address the third of our original questions – in addition to hastening larval development, exposure to male pheromones also delays the onset and shortens the duration of the post L3 lethargus, thus extending the feeding period. The feeding fraction (number of episodes of feeding divided by total number of recorded episodes for all animals) was 13% greater on MCP between 34 and 36 h (Fig. 3A). During this time the MCP larvae became ∼10% larger than the paired controls (Fig. 1E).

We concluded that, although the MCP and control larvae entered L4 almost simultaneously (Fig. 3C; Fig. S3A), the former started the L4 better provisioned and therefore capable of faster execution of developmental events in early L4. We saw no developmental acceleration until ∼36 h or early L4 (Fig. 2). By mid-late L4 the MCP advantage increased to ∼2 h (worms on MCP at 42 h almost equivalent to control at 44 h, Fig. 2A). The earlier onset of spermatogenesis (Fig. 2G) and fewer mature sperm (Fig. 2H) are consistent with this view, because these processes take place early in L4 (Ellis and Schedl, 2007). We speculate that larvae on MCP may be able to progress faster through the earliest portion of L4 when molting and developmental timers become synchronized (Meeuse et al., 2020; Romero-Expósito et al., 2024 preprint).

### A window of sensitivity to male pheromones during the late L3 stage

To determine when larval hermaphrodites needed to experience male pheromones to accelerate growth and development, we transferred them between MCP and control plates at different ages and for different periods of time. Only those treatments that included exposure during late L3 (between 32 and 36 h) accelerated development (Fig. 3E; Fig. S3C). Exposure to male pheromones only during this ∼4 h period in late L3 did not appear to accelerate development, but we note that at this age interindividual variability (Faerberg et al., 2021; Filina et al., 2022; Mata-Cabana et al., 2022; Stojanovski et al., 2022) is likely >4 h (Fig. 1A), making this result difficult to interpret. Late L3 was previously implicated in regulation of growth (Rai and Rankin, 2007) and in modulation of synaptic transmission (Qian et al., 2021) in response to social signals. We inferred that sensory inputs during a critical time window in late L3 modulate developmental events in L4.

### Coordination of larval development, behavior and environmental sensitivity

**Fig. 4.**
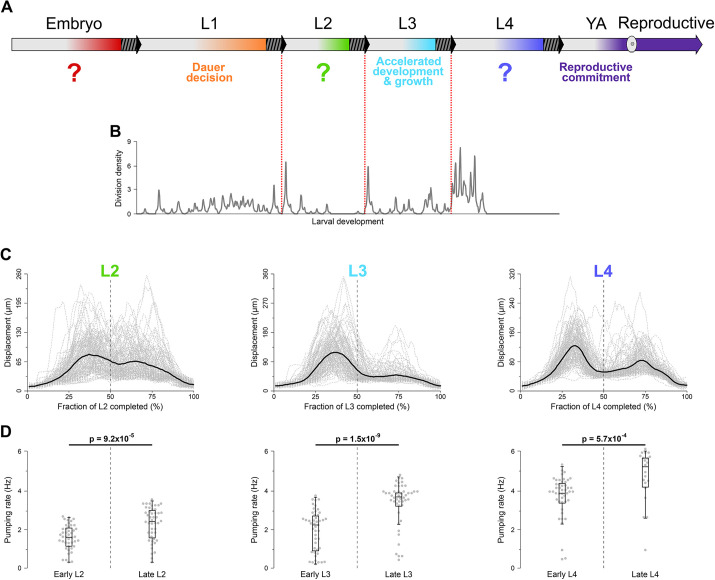
**Coordination of larval development, behavior and environmental sensitivity.** (A) Schematic of *C. elegans* life history. Striped-grey blocks and arrows represent lethargus and ecdysis. Color represents periods of environmental sensitivity that modulate development during the next stage. The question marks indicate largely unknown sensory inputs and their developmental consequences. (B) Density plot of post-embryonic cell divisions based on the classical lineage (Sulston and Horvitz, 1977). Additional data in Fig. S4. (C) Exploratory activity, inferred from patterns of locomotion, during L2, L3 and L4 larval stages. Re-plotting of original data reported by Stern et al. (2017). Dashed grey lines represent displacement over the previous 10 s by individual worms. Black lines represent population averages. (D) Pharyngeal pumping rates in the first and second halves of L2, L3 and L4. Each dot represents one individual. Box plot shows median values (middle bars) and first to third interquartile ranges (boxes); whiskers indicate approximately 1.5× the interquartile ranges. See Table S1 for sample sizes and statistical analyses.

We believe that our results offer provocative insights beyond those summarized in the three sections above. Evidence suggests that *C. elegans* developmental stages other than L3 may contain periods of sensitivity to environmental signals (Fig. 4A). Famously, under adverse conditions larvae can opt out of reproductive development to become dauer (Cassada and Russell, 1975), in part prompted by pheromones (Butcher et al., 2007; Srinivasan et al., 2008). The decision to enter dauer can only be made during mid/late L1 before the onset of L2 (Schaedel et al., 2012). Embryos in late stages of embryogenesis sense their environment to modify development in L1 (Bayer et al., 2022). Experiences during L2 impact developmental phenotypes in L3 (Braendle and Felix, 2008). During a short window early in adulthood, just before egg laying, hermaphrodites are particularly sensitive to food and male pheromones (Aprison et al., 2022).

Windows of environmental sensitivity tend to occupy the latter portions of developmental stages (Fig. 4A) and typically involve perception of food. They may be related to programs that regulate starvation arrests early in all larval stages (Baugh and Hu, 2020) and act as checkpoints to prevent development in the absence of food (Schindler et al., 2014). Starvation checkpoints represent regulated decisions made based on sensing the environment (Fukuyama et al., 2015), not the inability to continue development due to insufficient resources (Fukuyama et al., 2006; Schindler et al., 2014). Although food is a major determinant of developmental progression, other environmental signals, including social cues as we have shown here, are also important.

During periods of environmental sensitivity, animals gather information relevant to making decisions regarding major developmental events. One such event is molting at the end of each larval stage (Lazetic and Fay, 2017). Bursts of cell divisions occur in the beginning of larval stages, particularly L2, L3 and L4 (Fig. 4B; Fig. S4), and are among the developmental events that are arrested at checkpoints if adequate food is absent (Schindler et al., 2014). Recent studies demonstrated that expression of several thousand genes oscillates during larval development (Hendriks et al., 2014; Meeuse et al., 2020) and that nutrient sensing regulates rhythmic gene expression (Stec et al., 2021). We propose that during environmentally sensitive periods in each larval stage worms collect information on nutrient availability as well as social milieu and probably other features of the environment. Sensory inputs may adjust oscillating gene expression and alter the implementation of developmental decisions.

Certain behavioral states (Flavell et al., 2020), particularly food-associated behavior, may preferentially occur during environmentally sensitive periods, because nutrient sensing is a major aspect of environmental sensing. *C. elegans* roam (high-velocity movements with few turns) and dwell (slower movement with more frequent turns and reversals) on food (Ben Arous et al., 2009; Flavell et al., 2013; Fujiwara et al., 2002; Shtonda and Avery, 2006). Larvae tend to roam in early and dwell in late L3 and L4 stages (Stern et al., 2017). Both behavior and development may be regulated differently during L1, when worms show different behaviors under fed (Stern et al., 2017) and starved (Ali Nasser et al., 2023) conditions. The patterns of oscillating gene expression (Meeuse et al., 2020), growth (Nyaanga et al., 2022; Stojanovski et al., 2022) and development (Fig. 4B) in L1 also appear to be different than during other stages.

The periods of environmental sensitivity we propose (Fig. 4A) correspond well with periods of reduced locomotion (Fig. 4C). Patterns of stereotyped movement originate before the onset of larval development, during embryogenesis (Ardiel et al., 2022). In what is either a coincidence or a yet-to-be understood connection, oscillations in gene expression (Meeuse et al., 2020) and stereotyped movement during embryogenesis (Ardiel et al., 2022) are initiated around the same time (∼400 min post fertilization), approximately halfway through embryogenesis (Sulston et al., 1983).

Roaming is associated with exploration, whereas dwelling with resource exploitation (Ben Arous et al., 2009; Flavell et al., 2013; Shtonda and Avery, 2006). Consistent with the idea of coordinated motor programs (Cermak et al., 2020), we found that, during earlier portions of larval stages, worms had lower rates of pharyngeal pumping, a proxy for food intake (Fang-Yen et al., 2009) (Fig. 4D). We interpret distinct patterns of locomotion and feeding during periods of environmental sensitivity in light of the maximally informative foraging model (Calhoun et al., 2014). At the beginning of larval stages worms roam, broadly exploring their habitats to optimize food, social signals, etc. The ticking developmental clock inexorably shifts behavior toward dwelling, i.e., exploitation of resources, near the site where developmental events at the beginning of the next larval stage will unfold. Worms interpret sensory inputs obtained during the latter parts of larval stages to modulate development in ways best suited for the locale. This may range from developmental arrest if the conditions are poor to accelerated development in the presence of potential mates.

Distinct behavioral epochs within larval stages could be detected even under the replete and stable conditions of laboratory culture. In patchy, ephemeral environments of the natural *C. elegans* habitats (Schulenburg and Felix, 2017), the distinctions between behavioral patterns in the early versus late portions of a larval stage may be more pronounced and may extend beyond the locomotion, food consumption and cue sensing behaviors addressed here.

### Parallels in other species

Phenomena similar to the ones described here have been documented in other species. In *D. melanogaster*, larvae of different ages exhibit different patterns of locomotory behavior (Sokolowski et al., 1984). High-resolution long-term monitoring promises to expand our understanding of behavior (Elliott et al., 2021). In *Drosophila*, continuous monitoring of larval crawling on timescales comparable with the duration of one larval instar revealed apparent epochs of faster directional movement with fewer turns followed by prolonged periods of slower movement with more turns (Yu et al., 2023), a pattern strikingly similar to the one observed during long-term monitoring of *C. elegans* larvae (Stern et al., 2017) (Fig. 4C). *Drosophila* larvae display characteristic patterns of activity associated with cessation of feeding at the end of larval development (Wegman et al., 2010). Such association between feeding behavior and developmental progression likely reflects the requirement to attain ‘critical weight’ to initiate metamorphosis (Nijhout, 2003). The coupling between growth/development and behavioral states may be direct – in *Drosophila*, bouts of locomotor activity acutely inhibit insulin-producing cells (Liessem et al., 2023). We conclude by emphasizing that although nutrition has a major impact on larval development and behavior, other environmental variables (e.g. Callier and Nijhout, 2011), including social signals as we demonstrated here, are also important. In most cases, specific roles of various signals, their integration, and mechanisms by which they alter behavior and development, remain to be discovered.

## MATERIALS AND METHODS

### *C. elegans* handling and strains

*C. elegans* nematodes were maintained using standard methods (Brenner, 1974) on nematode growth media (NGM) plates seeded with OP50 *E. coli*. Unless otherwise noted, all experiments were carried out at 20°C with synchronized N2 hermaphrodites grown in isolation (1 worm/plate). In this study we measured time from release from the L1 arrest. Previously, we demonstrated that developmental acceleration on MCPs was observed regardless of whether larvae were synchronized by egg preparations or timed egg lay (Aprison and Ruvinsky, 2016). Although developmental schedules in dozens of experiments presented here were highly consistent, they occasionally differed (by 1-2 h) from others presented elsewhere (for example, Sulston and Horvitz, 1977). These subtle differences could be due to slightly different incubation temperature, different bacterial food, greater population density commonly used when maintaining worm cultures or other differences in the details of experimental procedures. We carried out experiments with paired controls to ensure appropriate comparisons.

Synchronous worm cultures were obtained by bleaching gravid adults with an alkaline hypochlorite solution and hatching embryos overnight (≤16 h) in M9 (Sulston and Hodgkin, 1988). Arrested L1 larvae were plated (20-30 worms/plate) onto 60 mm lawn plates of OP50. Immediately after, worms were singled onto 35 mm plates that were seeded with 5 μl of 1:10 OP50 dilution and grown overnight. MCP were made by placing a single young adult N2 male for 24 h and removing it before the start of the experiments. The following strains were used: N2 wild type, SV1009 heIs63 [*wrt-2*p::GFP::PH+*wrt-2*p::GFP::H2B+*lin-48*p::mCherry] (Wildwater et al., 2011), PD4667 ayIs7 [*hlh-8*::GFP fusion+*dpy-20*(+)] (Harfe et al., 1998), NK881 [qyIs166[*cdh-3*>GFP::CAAX]; qyIs127[*lam-1*::mCherry] (Naegeli et al., 2017).

### Imaging progression of developmental events

For all imaging, except for body volume, worms were mounted onto 2% agarose slides, observed and imaged on a Leica DM5000B microscope using a Retiga 2000R camera. L4 vulva substages were scored manually based on the morphology of the vulval lumen. Standard definitions (Mok et al., 2015; Seydoux et al., 1993) were adapted to the specifics of our experimental procedures to derive a consistent series of substages. L4.0 could be distinguished from the late L3 by observing the shed cuticle; L4.1, vulva undergoes invagination; L4.2, invagination progresses beyond the ventral P6.p great-granddaughter cells; L4.3, convex sides develop. L4.4, upside-down T shape with sharp corners forms; L4.5, rounded corners and ‘fingers’ develop; L4.6, ‘fingers’ start pointing ventrally; L4.7, vulva starts collapsing forming a maple leaf shape; L4.8, vulval collapse progresses leaving a small invaginated space; L4.9. vulval lips protrude outwards but remain covered by the cuticle. See Fig. S2A for representative images.

The extent of germline development (Fig. 1B,C) was scored as the number of oocytes completely spanning the gonad lumen and the number of fertilized embryos in the uterus.

To estimate body volume (Fig. 1D,E) worms were imaged without removal from 35 mm NGM plates. The images were taken using an Olympus SZ61 stereomicroscope fitted with a Lumenera Infinity 2 camera and processed manually using ImageJ. Length was measured using the segmented line tool by skeletonizing the worm following the midline from most anterior to most posterior discernable points. Width was measured at the level of the vulva, if identifiable, or at ∼2/3 of body length. Volume was estimated assuming cylindrical shape of the larvae [V=π×length×(width/2)^2^]. The image in Fig. S1D shows skeletonization used for ImageJ processing.

Progression of certain developmental events in the soma was ascertained using reporter strains. Seam cell development (Fig. 2A) was monitored using the SV1009 strain (*wrt-2*::GFP) that helped to visualize divided and adhered cells. The H and V blast cells and their progeny undergo cell divisions at the beginning of L2, L3, and L4 stages to give rise to the hypodermal seam cells; adhesion occurs later in the stages (Podbilewicz and White, 1994). See Fig. S2B for representative images.

Sex myoblast divisions (Fig. 2B) were scored in the PD4667 strain (*hlh-8*::GFP). Sex myoblast cells are the progeny of the M blast cell, which comes from a different founder cell (MS) than the H and V blast cells (AB) (Sulston et al., 1983). See Fig. S2C for representative images used for staging.

Morphological transformations during invasion of the vulva anchor cell (AC; another descendent of the MS lineage) (Fig. 2C) was scored in the NK881 strain (*cdh-3*::GFP). Staging (see Fig. S2D for representative images) was based primarily on the shape on the ventral side of the cell broadly following Sherwood and Sternberg (2003). AC1, long curving ventral side not attached to P6 daughter cells; AC2, flat and short ventral side indicates attachment; AC3, invasive protrusion forms a V-shape; AC4, invasive protrusion retracts forming an M-shape. See Fig. S2D for representative images.

During late larval development, the hermaphrodite gonad changes considerably (Fig. S2E) (Kimble and Hirsh, 1979). In addition to growth, the gonad undergoes characteristic morphological changes (Antebi et al., 1998; Hedgecock et al., 1987; Kimble and Hirsh, 1979). Gonad length was measured by following the midline of the gonad using the segmented line tool in ImageJ if the entirety of the gonad was visible. Turning phases were defined as follows: TP1, gonad and distal tip cell (DTC) are extending along the ventral side away from the vulva; TP1/2, gonad is continuing to extend, DTC has initiated a dorsal turn; TP2, gonad and DTC extend dorsally; TP2/3, DTC begins to extend along the dorsal side toward the midline defined by the vulva. See Fig. S2E for representative images.

Starting in mid to late L3, the population of germline progenitor cells rapidly expands (Hubbard and Schedl, 2019). For counting nuclei in the progenitor zone [for definition, see Crittenden et al. (2006)], hermaphrodites were stained with DAPI (4′,6-diamidino-2-phenylindole) using a previously described (Aprison and Ruvinsky, 2016) variation of a published protocol (Pepper et al., 2003). In addition to mitotic nuclei, this population contains some nuclei in the early stages of meiosis (Fox et al., 2011). For sperm counts, 54 h hermaphrodites were stained with DAPI as above. The antibody that recognizes minor sperm proteins (SP56) is an early marker of sperm differentiation (Ward et al., 1986), a process that begins in early L4 (Lamont and Kimble, 2007).

### Immunohistochemistry

Worm dissection and antibody staining were modified from a published protocol (Crittenden et al., 2017). At 42, 44 and 46 h control and MCP hermaphrodites were picked into 30 μl PBS-0.1% Tween 20 with 0.25 mM levamisole in the bottom half of a large glass Petri dish. Hermaphrodites were cut with a scalpel to extrude the germline, and ∼30 animals per condition were dissected in ∼5 min. The dissected animals were transferred to a 1.5 ml microcentrifuge tube and incubated with 3% paraformaldehyde in PBS-Tween for 30 min at 20°C with rocking. The paraformaldehyde was washed off and the worms were fixed in −20°C methanol overnight. The methanol was washed off and the worms were blocked with 3% bovine serum albumin in PBS-Tween for 30 min at 20°C with rocking. The blocking agent was washed off and the worms were incubated overnight with the primary antibody [anti-SP56 antibody (Ward et al., 1986), a gift from the Kimble lab, diluted 1:50 in block] at 4°C with rocking. The following morning the worms were washed 3× with PBS-Tween at 20°C with rocking (≥10 min) and subsequently incubated with the secondary antibody (goat anti-mouse IgG H&L Alexa Fluor® 488, Abcam, 150113, diluted 1:1000 in PBS) for 2 h at 20°C with rocking. The worms were washed again 3× with PBS-Tween as above, suspended in 12 μl Vectashield with DAPI, and transferred to 2% agarose pads. Imaging was performed as above.

### Scoring pumping at the L3-to-L4 transition

For these experiments, worms were synchronized using a timed (1 h) egg lay and singled as early L1s. At 32 h, singled larvae were removed from 20°C and continuously observed until L4 (39 h) at room temperature. For each worm, grinder movement was observed for 10 s every 20 min on a Leica MZ16 stereomicroscope. Worms were categorized as L3 if active pumping was observed, as quiescent if no pumping was observed, or L4 if pumping and an invaginated vulva were observed. To limit bias, we scored five worms from control plates, followed by five MCP worms, until all worms were examined.

### Obtaining the density plot of timing of post-embryonic cell divisions

The unlabeled *C. elegans* lineage diagram was obtained from WormAtlas (https://www.wormatlas.org/images/lineage.png) and cropped to include only post-embryonic divisions [final size: 1034×2317 pixels (px), height and width, respectively]. The image consisted of three types of elements: vertical lines with width=2 px and height >2 px; horizontal lines with width >2 px and height=2 px; and ‘X’ markers consisting of five elements with height and width of each ≤2 px. As horizontal lines represented division events, we used a Python script to scan the image top-down by row (increasing *y*-coordinate, aligned with time progression) and deleted all elements with width ≤2 px. The processed image was then scanned by row again to count the number of contiguous regions of black pixels (i.e. discrete horizontal bars), each representing a division event. Sharp peaks appearing at coordinates 448-449 and 684-685 corresponded to seam cell divisions. These events occurred shortly after the onset of L2 and L3 at ∼16 and ∼25 h post hatching, respectively. The knowledge of this timing allowed us to convert the time scale from the image *y*-coordinate (row) to hours of development. Division density was then calculated using an inverse kernel: for value *V*_*i*_ density 
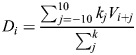
, where weight 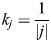, unless *j*=0 when *k*_*j*_=1. The procedure is shown schematically in Fig. S4. The code used to process the post-embryonic lineage diagram was deposited at: https://github.com/denisfaer/Faerberg_et_al_2023_Acceleration/blob/main/plotscan.py.

### Generating activity profiles

The original data on locomotion of 125 N2 worms throughout larval development were collected by Stern et al. (Stern et al., 2017). Durations of larval stages inferred from these data have been reported (Faerberg et al., 2021). For each individual's larval stage *j* activity profile point, 
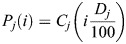
, where *i* is the percentage of stage completion, *D*_*j*_ is the duration of the individual's stage *j*, and *C*_*j*_(*t*) is the individual's activity curve as inferred (Faerberg et al., 2021) and cropped to larval stage *j*. The average of *P*_*j*_(*i*) for all individuals for each *i* yielded the population averages plotted in Fig. 4C. The code used to generate these profiles was deposited: https://github.com/denisfaer/Faerberg_et_al_2023_Acceleration/blob/main/stageact.pas.

### Quantifying the pharyngeal pumping rate

At 19 h (first half of L2), 23 h (second half of L2), 28 h (first half of L3), 34 h (second half of L3), 41 h (first half of L4) and 46 h (second half of L4), 50 worms reared on control plates were recorded for ∼10 s on a Leica MZ16 stereomicroscope. For each worm, the number of grinder movements was manually counted and normalized by the duration of the recording in seconds. Worms with no observed grinder movements or those located off the bacterial lawn were excluded from the analysis.

### Statistical analyses

Tests of statistical significance were carried out in R and Excel. Sample sizes and *P*-values are shown in Table S1.

## Supplementary Material

10.1242/develop.202961_sup1 Supplementary information

Table S1 (primary data and statistics)
