# Supplementary material for: Accelerated hermaphrodite maturation on male pheromones suggests a general principle of coordination between larval behavior and development
Source: Development. 2024 Jul 8;151(13):dev202961. doi: 10.1242/dev.202961 (PMC11266794; doi:10.1242/dev.202961)
Supplement: Supplementary information [file develop-151-202961-s1.pdf]

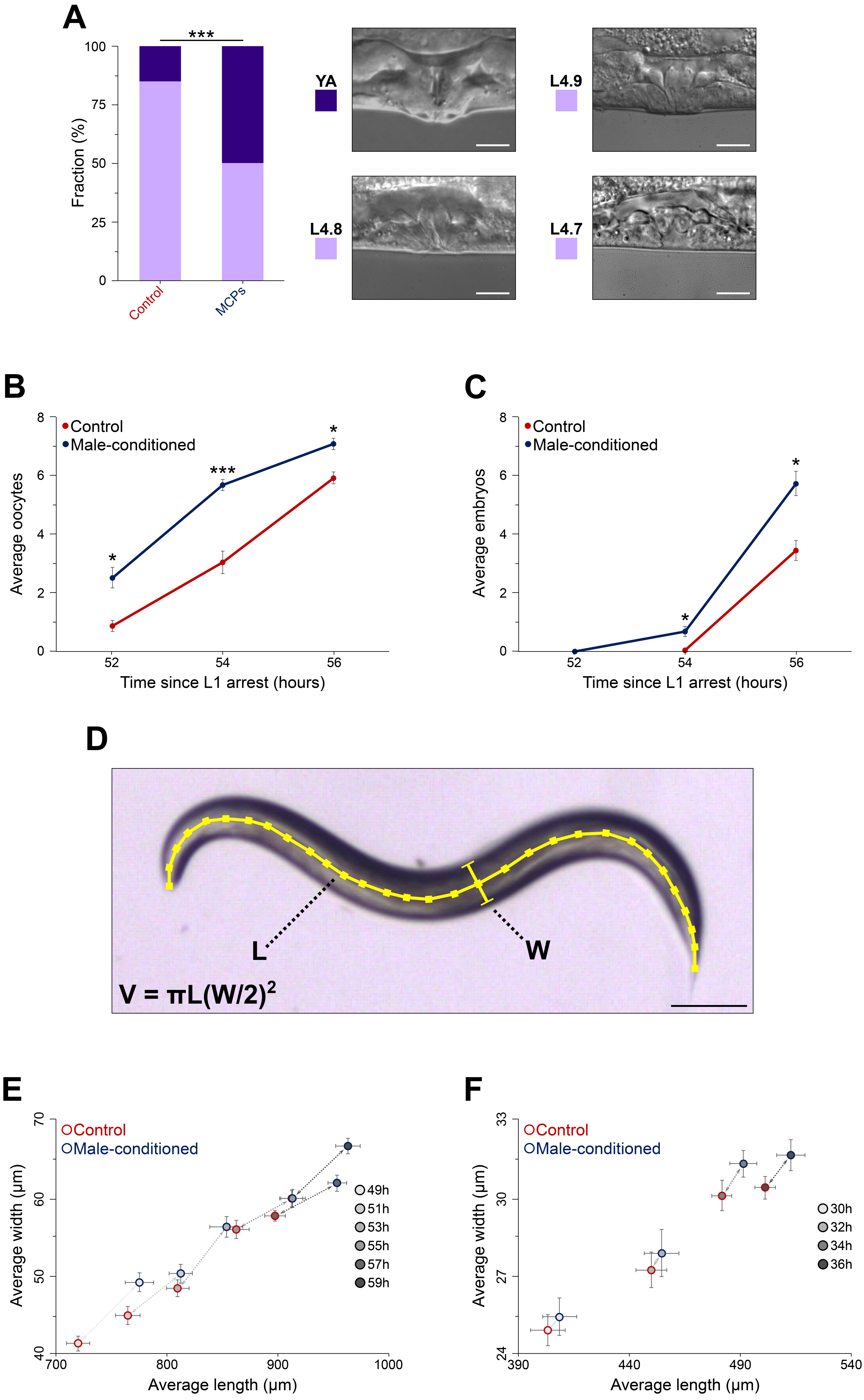
A
***
100
75
50
25
0
Fraction (%)
Control
MCPs
YA
L4.9
L4.8
L4.7
B
8
6
4
2
0
Control
Male-conditioned
*
***
*
Average oocytes
52
54
56
Time since L1 arrest (hours)
C
Control
Male-conditioned
*
*
Average embryos
52
54
56
Time since L1 arrest (hours)
D
L
W
V = πL(W/2)²
E
70
60
50
40
Control
Male-conditioned
49h
51h
53h
55h
57h
59h
Average width (μm)
700
800
900
1000
Average length (μm)
F
33
30
27
24
Control
Male-conditioned
30h
32h
34h
36h
Average width (μm)
390
440
490
540
Average length (μm)

Fig. S1. Male pheromones accelerate development and growth of hermaphrodite larvae. Related to Figure 1.

(A) Distributions of morphological vulva substages (as defined on the right; also Figure S2A) on control and male-conditioned plates at 50 hours post release from L1 arrest. **(B)** Related to Figure 1B. Average number of oocytes spanning the gonad lumen. Whiskers show standard error. **(C)** Related to Figure 1C. Average number of embryos in the uterus. **(D)** Related to Figure 1D, E. Sample worm image used to estimate body volume. Yellow lines indicate the midline (length, L) and cross-section (width, W) for ImageJ processing. Scale bar = 100 µm. **(E)** Related to Figure 1D. Average length and width of worms emerging into adulthood. Circle color indicates growth condition, circle fill color indicates age, dashed arrows connect the same timepoint in control and MCP groups. Whiskers show standard error. **(F)** Related to Figure 1E. Average length and width of worms in the second half of the L3 stage. Symbol meaning is as in Figure S1E. See Table S1 for sample sizes and statistical analyses.

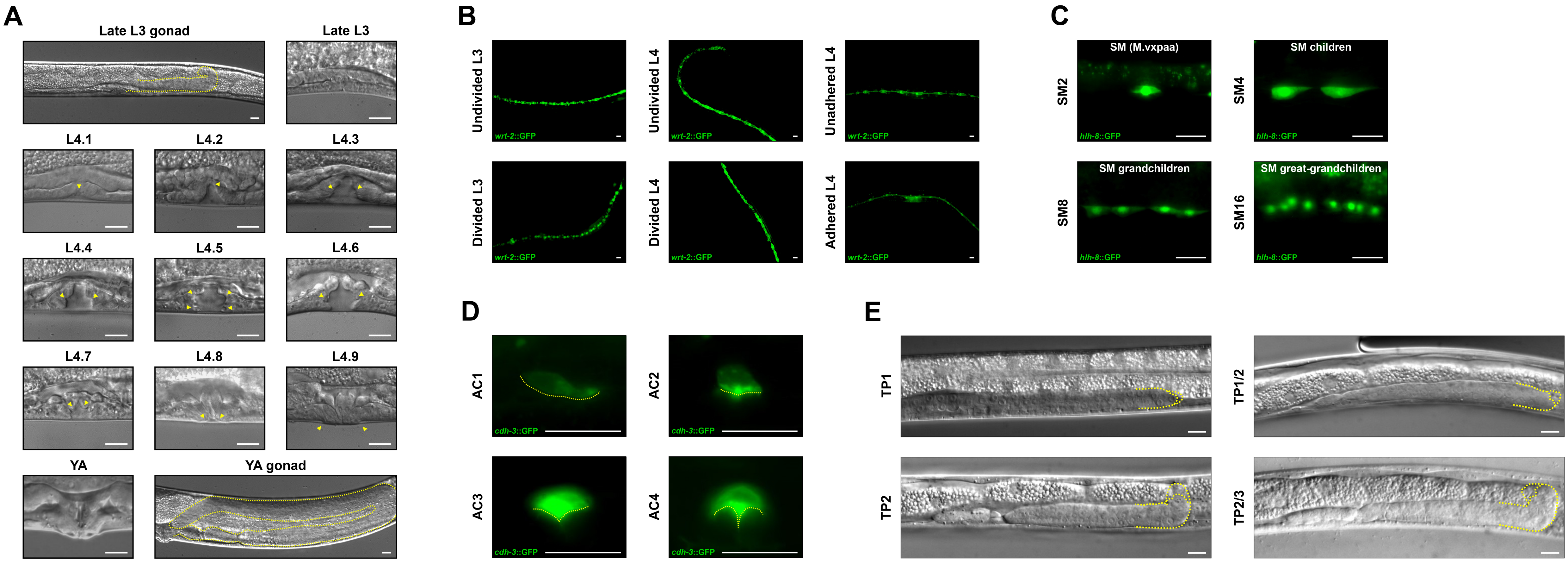

Fig. S2. Morphological definitions of scoring criteria.
(A) Morphological features of the vulva and the gonad during different substages in late hermaphrodite larvae. Based on previously described definitions (Mok et al., 2015; Seydoux et al., 1993) and adapted to our experimental procedures. See Materials and Methods for stage descriptions. Dashed yellow outlines show gonad progression, yellow arrowheads point to key morphological features. **(B)** Fluorescence microscopy images of seam cells (*wrt-2*::GFP) in representative individuals before (top) and after (bottom) undergoing early-L3 division (left), early-L4 division (middle), and mid-L4 adhesion (right). **(C)** Fluorescence microscopy images of sex myoblasts (*hlh-8*::GFP) undergoing three rounds of divisions. **(D)** Fluorescence microscopy images of the invading anchor cell (*cdh-3*::GFP). See Materials and Methods for stage descriptions. **(E)** Representative DIC images of mid- to late-L3 individuals that show the phases of gonad turning. See Materials and Methods for stage descriptions. Dashed yellow outlines show the distal portion of the gonad and DTC orientation. All scale bars = 10μm.

Mok, D. Z., Sternberg, P. W. and Inoue, T. (2015). Morphologically defined sub-stages of C. elegans vulval development in the fourth larval stage. BMC Dev Biol 15, 26.

Seydoux, G., Savage, C. and Greenwald, I. (1993). Isolation and characterization of mutations causing abnormal eversion of the vulva in Caenorhabditis elegans. Dev Biol 157, 423-436

Development • Supplementary information

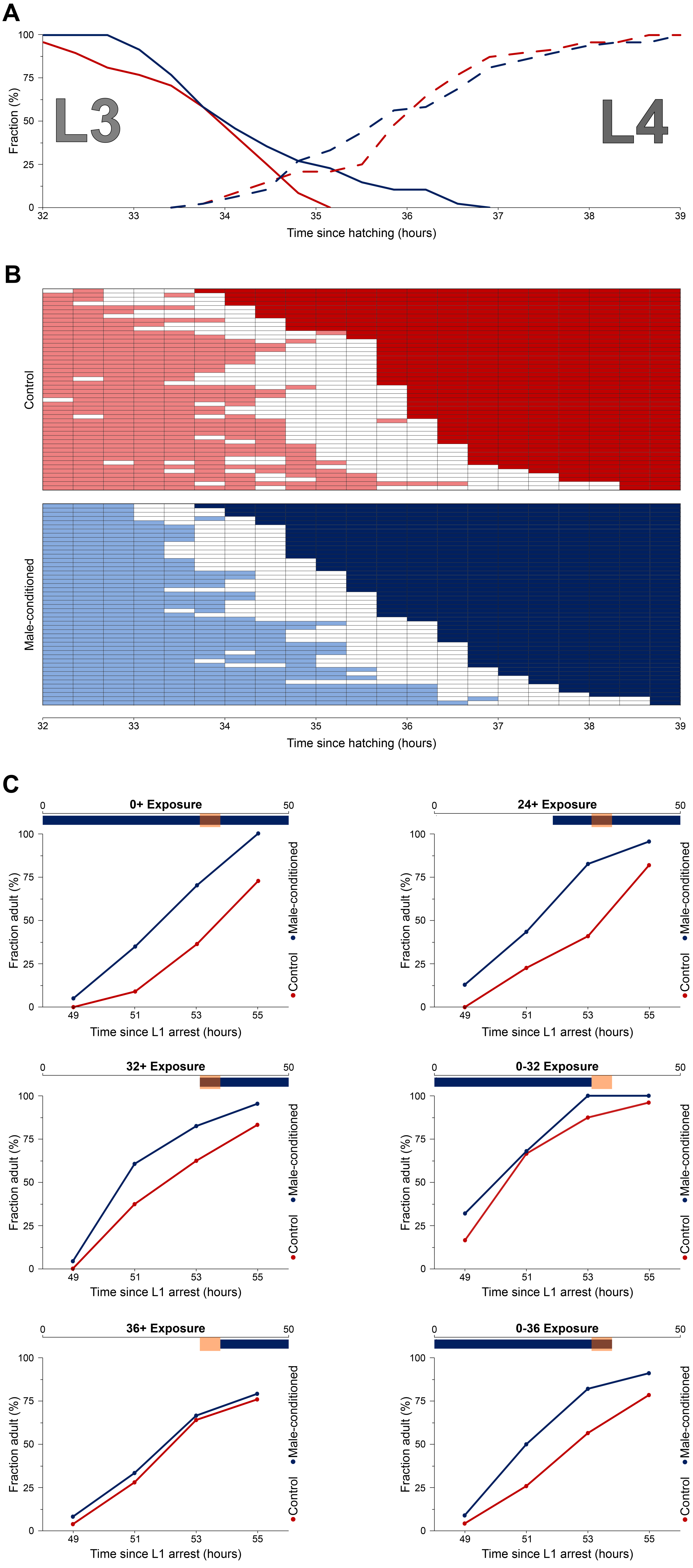

Development • Supplementary information

Fig. S3. Male pheromones and developmental timing. Related to Figure 3.

(A) Fractions of 48 individuals (data shown in Figure 3A) in L3 (solid lines) and L4 (dashed lines). Blue = MCP, red = control. **(B)** The data presented in Figure 3A rearranged in order of the onset of L4. This view highlights the instances of larvae stopping pharyngeal pumping, then re-initiating, and then stopping again for the duration of lethargus. This particularly noticeable in control worms. **(C)** Representative results of experiments summarized in Figure 3F. Periods of exposure to male pheromones are indicated on top of each sub-panel with a thick blue line and numerical notation. Orange boxes correspond to the period between 32 and 36 hours. In the “0-32 Exposure”, both the treated and control samples developed somewhat faster than in other experiments. Due to occasional occurrence of this kind of systematic variation, likely due to external environmental conditions like temperature, we always compared treated samples to paired controls. See Table S1 for sample sizes and statistics.

Development • Supplementary information

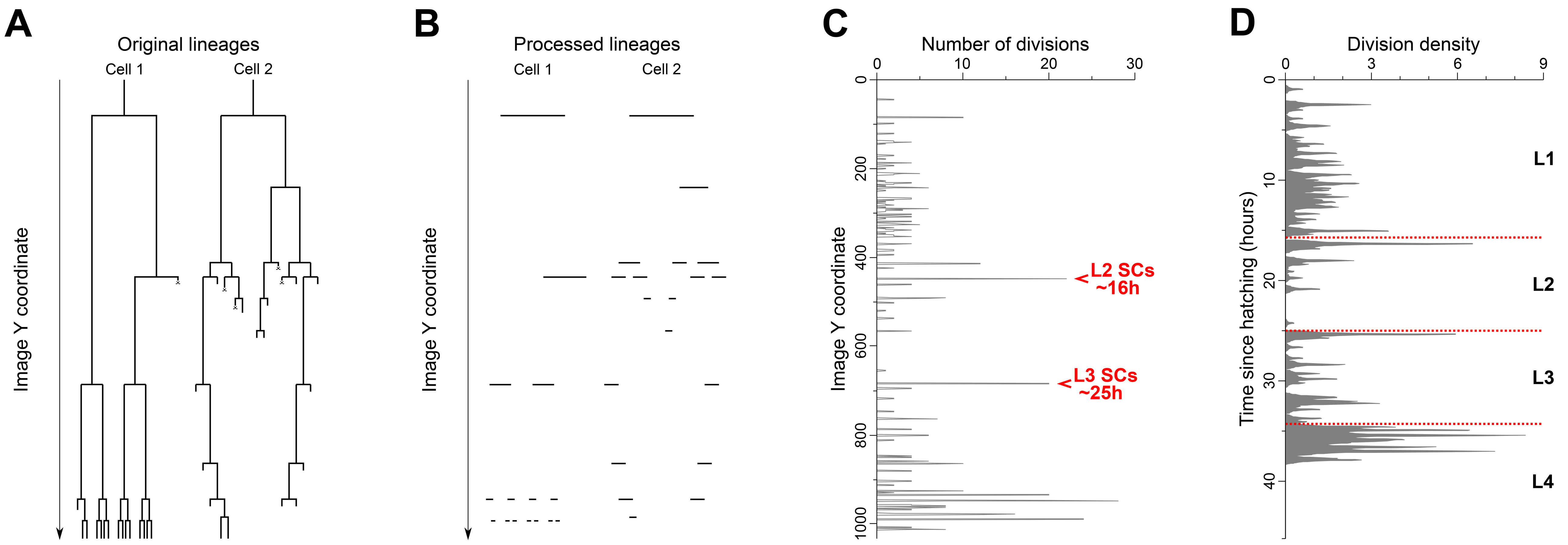

Fig. S4. Obtaining the density plot of timing of post-embryonic cell divisions. Related to Fig. 4.

(A) A hypothetical lineage tree similar to the post-embryonic lineage diagram of *C. elegans*. **(B)** Image processing to infer division times in the tree shown in panel A. Processing removed all objects in every row with width ≤ 2 pixels but retained horizontal lines representing divisions. Removed items included vertical lines and “X” markers representing cell deaths. Scanning each row and counting contiguous blocks of black pixels yields the number of cell division events. Panels A and B are shown for the purpose of illustrating the approach. **(C)** Unlike the hypothetical schematics in panels A and B, this panel shows analysis of actual division events during *C. elegans* post-embryonic development as given in the classical lineage diagram (Sulston and Horvitz, 1977) (https://www.wormatlas.org/images/lineage.png). Inferred number of divisions (calculated as described in B) at each timepoint (the Y axis denotes time). Marked spikes in rows ~450 and ~680 are due to L2 and L3 Seam Cell divisions occurring at ~16 and ~25 hours post hatching. These landmarks allow a conversion from a time scale expressed in “rows” to a time scale in absolute hours of development. **(D)** A kernel density plot of the data from panel C visually represents some inter-individual variability in division timing. The time scale was converted into hours, as described above. Dashed red lines demarcate boundaries between larval stages. More details in Materials and Methods.

Sulston, J. E. and Horvitz, H. R. (1977). Post-embryonic cell lineages of the nematode, Caenorhabditis elegans. *Dev Biol* **56**, 110-156.

Table S1 (primary data and statistics)

Available for download at
https://journals.biologists.com/dev/article-lookup/doi/10.1242/dev.202961#supplementary-data

Development • Supplementary information
